# Spinal paraganglioma at the conus medullaris mimicking schwannoma: A case report

**DOI:** 10.1016/j.ijscr.2022.107698

**Published:** 2022-09-24

**Authors:** Mohammed Maan Al-Salihi, Muath Hussein, Maryam Sabah Al-Jebur, Sabrina Rahman, Ali Ayyad, Md Moshiur Rahman

**Affiliations:** a Department of Neurosurgery, Hamad General Hospital, Doha, Qatar; b College of Medicine, University of Baghdad, Baghdad, Iraq; c Department of Public Health, Independent University-Bangladesh, Dhaka, Bangladesh; d Department of Neurosurgery, Saarland University Hospital, Homburg, Germany; e Neurosurgery Department, Holy Family Red Crescent Medical College, Dhaka, Bangladesh

**Keywords:** Conus medullaris, Paraganglioma, Spine

## Abstract

**Background:**

Paragangliomas of the spine are extremely rare, and they should be considered in the differential diagnosis of spinal tumors due to its overlapping clinical and radiological features with many spinal tumors.

**Case report:**

In this article, we present a 30-year-old lady who presented with low back pain and radicular neuropathic pain at L1 dermatome which was intractable to medical surgery. Her magnetic resonance imaging (MRI) of the lumbosacral spine revealed a T1 isointense, T2 heterogeneously hyperintense intradural extramedullary lesion at the conus medullaris with strong homogenous enhancement on contrast administration. The lesion was surgically excised completely with L1 laminectomy, and the histopathological picture was suggestive of paraganglioma. The patient's complaints resolved fully postoperatively, and there was no evidence of recurrence on long-term follow-up.

**Conclusion:**

Due to the absence of pathognomonic clinical or radiological features of paragangliomas, they should be taken into consideration in the differential diagnosis of spinal tumors. They share similar clinical and radiological features of schwannomas, ependymomas, and hemangioblastomas. The diagnosis is usually made postoperatively based on histopathological examination.

## Highlights

• Paragangliomas overlap the clinical and radiological features with other tumors.
• Diagnosis is made postoperatively based on histopathological examination.
• It should be considered in the differential diagnosis of spinal tumors.

## 1 Introduction

Paragangliomas are rare tumors originating from neuroendocrinal cells dispersed throughout the body [1]. The largest cluster of neuroendocrine cells is located within the adrenal medulla, and smaller clusters are located in the head, neck, and paravertebral regions [1]. The paraganglia are essentially two types: sympathetic paraganglia and parasympathetic paraganglia [1]. Paragangliomas are either intra-adrenal (i.e., pheochromocytomas) or extra-adrenal autonomic (sympathetic or parasympathetic) paragangliomas [2]. They are classified according to their anatomical location and secretory function [2]. Anatomically, paragangliomas commonly originate at the carotid body, jugular ganglion, jugulotympanic ganglion, or other ganglia in the head and neck region (e.g., laryngeal ganglia, nodose ganglion, … etc.) [3]. Less commonly, paragangliomas originate at aorticopulmonary ganglion, pre-aortic ganglia, or ganglia of the sympathetic trunk [3]. Paragangliomas are rare in the spinal canal and, when they occur, they are found in the extramedullary intradural compartment of the lumbosacral region [4]. Based on their function, paragangliomas are classified into functioning (i.e., catecholamine-secreting) and non-functioning tumors [5].

Paragangliomas are rare tumors occurring in approximately 0.8 per 100,000 population per year [6]. They affect males and females equally, and commonly present between the third to the fifth decades of life [5]. The vast majority of these tumors are sporadic [5]. However, up to one third of these tumors are associated with inherited syndromes (e.g., neurofibromatosis type 1, multiple endocrine neoplasia type 2, von Hippel Lindau syndrome, and Carney-Stratakin dyad syndrome) [6]. The clinical presentation of paragangliomas varies according to the tumor location and secretory function [5]. Symptoms of mass effect depend on the anatomical location of the tumor (and may remain asymptomatic for years or even decades), and catecholamine hypersecretion symptoms include hypertension, headache, diaphoresis, palpitation, dyspnea, and tremors [5].

In this article, we present a rare case of paraganglioma of the conus medullaris and discuss its presentation, differential diagnosis, diagnostic approach, treatment, and work-up.

## 2 Case report

This was a 30-year-old female who presented at the outpatient clinic with a three-month history of progressive low back pain and leg neuropathic pain. She reported paresthesia of both lower limbs predominantly on the right side reaching up to the inguinal regions. The paresthesia was more prominent at night, and it was refractory to medical therapy. She denied any weakness, bowel or bladder dysfunction, or upper limb affection. Her past history was unremarkable. She was born to non-consanguineous parents, and she had no family history of similar condition, related neurological diseases, or neoplasia. Her general physical examination was unremarkable. Her neurological examination revealed mild clasp-knife spasticity in both lower limbs (particularly on the right side), bilateral extensor plantar response, brisk knee and ankle reflexes, sensory level at L1 with involvement of the saddle-shaped area (for pin-prick sensations).

**Fig. 1:**
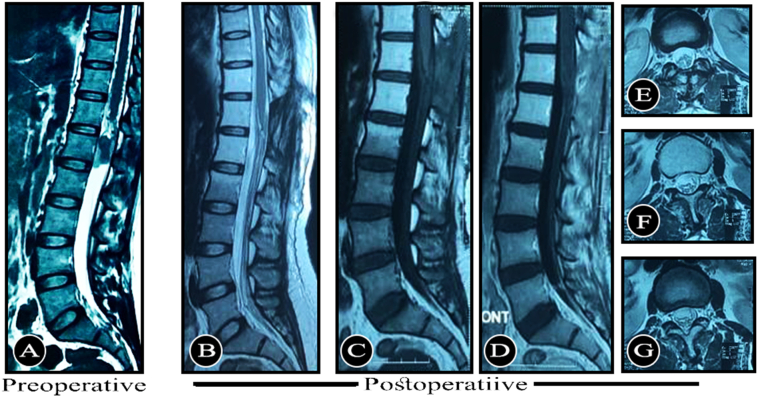
Pre- and post-operative MRI of the lumbosacral spine of the patient showing (a) Pre-operative T2 hyperintense lesion with heterogenous signal (B to G) Post-operative MRI showing successful removal of the lesion with L1 laminectomy with no postoperative residual on sagittal T2 (B), sagittal T1 (C), sagittal T1 with contrast (D), or axial T2films (E to G).

**Fig. 2:**
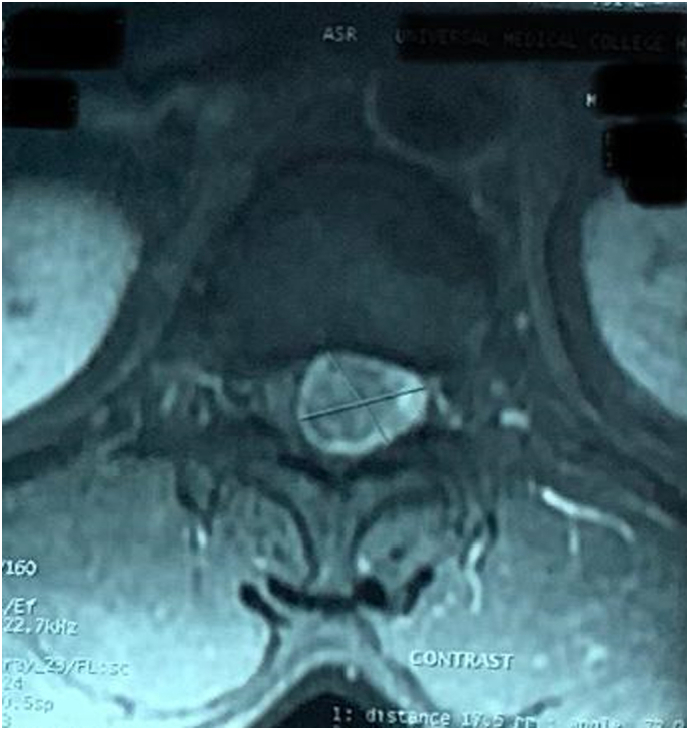
Contrast enhanced axial MRI showing the tumor.

The patient underwent lumbosacral magnetic resonance imaging (MRI) of the spine with contrast, and it revealed a T1 isointense, T2 hyperintense (Fig. 1A) intradural extramedullary lesion at the conus medullaris at L1 level with strong homogenous enhancement on contrast (Fig. 2) adminstration.

The patient underwent surgical laminectomy at L1, and the tumor was completely removed successfully without any residual (Fig. 1B to G). Histopathological examination by the hematoxylin and eosin (H&E) and immunohistochemistry of the excised mass revealed clusters of epithelioid cells arranged in a Zellballen pattern and separated by prominent fibrovascular stroma. The cells were oval to polygonal with abundant granular cytoplasm and large nuclei. There were no mitotic figures or features of nuclear atypia. The histopathological features were suggestive of paraganglioma. The postoperative follow-up was uneventful. The patient recovered completely, and she is pain-free to date.

## 3 Discussion

We presented a middle-aged lady who presented with a spinal cord syndrome that was found to be due to paraganglioma. Paragangliomas of the spine are rare extra-adrenal neuroendocrine tumors that have been rarely reported in the literature. Extra-adrenal paragangliomas occur in the head and neck region in >90 % of the cases [7]. In the nervous system, paragangliomas commonly occur in the Sella turcica, pineal gland, petrous ridge, or the spinal cord [8]. Based on the world health organization (WHO) most recent classification in 2021 [9], they are classified into cranial and paraspinal paragangliomas.

Paraspinal paragangliomas are extremely rare. They have an estimated annual incidence of approximately 0.07 per 100,000 population [10]. Cauda equina is the most common site reported for spinal cord paraganglioma. In our patient, the lesion occurred in the conus medullaris and was successfully treated with total surgical excision without evidence for recurrence on long-term follow-up. The clinical presentation and outcome were also similar to our case in many cases of cauda equina paragangliomas reported in the literature [11], [12]. Conus medullaris involvement was reported in fewer cases such as the case described by Diyora et al. [10] and three of the cases reviewed by Shtaya et al. [13]. In all these cases, the clinical presentation was only related to mass effect with no signs of catecholamine hyperactivity, and there was no evidence of long-term recurrence.

In other case series and literature reviews, the clinical presentations varied. For instance, Mishra et al. [8] reported eight cases with primary spinal paragangliomas who were diagnosed over a six-year period between 2008 and 2013. The patients' age ranged between 34 and 77 years, and the vast majority were males (6 out of the 8 patients) [8]. All the lesions occurred between D12 and L4 levels and presented with low back pain and sciatica [8]. Preoperatively, they were diagnosed as either schwannoma or ependymoma [8]. In another case series of 31 cases of cauda equina paraganglioma, Sonneland et al. [14] described the clinical features and immunocytology profile of 18 males and 13 females. Low back pain, combined motor sensory deficit, sensory deficits only, bowel/bladder dysfunction, and motor deficits only occurred in 87 %, 35 %, 13 %, and 6 % of cases, respectively [14]. Total surgical excision was the mainstay treatment in 26 cases [14]. Three cases underwent subtotal surgical excision, and two cases needed radiotherapy [14]. A tertiary center experience in London described 10 cases of cauda equina paraganglioma where low back pain was the presenting manifestation in 94 % and successful surgical resection without recurrence was achieved in 93 % of all cases [13]. Some cases were associated with other features. One case with cauda equina paraganglioma, reported by Steel et al. [15], had associated syringomyelia of the cervical thoracic spinal cord canal.

Though paragangliomas of the spine are relatively rare, they should be considered in the differential diagnosis of spinal tumors. Many of the spinal cord tumors, especially schwannoma and ependymoma, share similar clinical, radiological and histopathological features with paraganglioma. Accordingly, the differential diagnosis is challenging. Unless they present with catecholamine hypersecretion syndrome, there are no specific investigation that can confirm the diagnosis of paragangliomas preoperatively [13]. The MRI image of paragangliomas overlaps with many tumors such as schwannomas, ependymomas, and hemangioblastomas [12]. Whilst the presence of hemorrhage and/or cysts was suggested to be a differentiating feature suggestive of schwannoma, case reports of hemorrhage and cyst formation were also described in paragangliomas [15].

Total surgical excision is the gold standard treatment modality for spinal paragangliomas [9]. Though almost >90 % of cases achieve cure after surgery, a small proportion of patients might experience recurrence [13]. Recurrence is more common in patients who undergo subtotal resection, and postoperative radiotherapy did not seem to affect the recurrence rate [8].

## 4 Conclusion

Due to the absence of pathognomonic clinical or radiological features of paragangliomas, they should be taken into consideration in the differential diagnosis of spinal tumors. They overlap the clinical and radiological features with other tumors such as schwannomas, ependymomas, and hemangioblastomas. Definitive diagnosis of paragangliomas is usually made postoperatively on histopathological examination of the excised specimen.

## Consent

Written informed consent was obtained from the patient for publication of this case report and accompanying images. A copy of the written consent is available for review by the Editor-in-Chief of this journal on request.

## Ethical approval

Hospital exempts ethics approval for reported cases.

## Funding

None.

## Author contribution

All authors equally contributed to the analysis and writing of the manuscript.

## Guarantor

Md. Moshiur Rahman

## Research registration number

N/a.

## Declaration of competing interest

None.
